# Supplementary material for: Splice-Junction-Based Mapping of Alternative Isoforms in the Human Proteome
Source: Cell Rep. Author manuscript; Available in PMC 2020 Jan 15. (PMC6961840; doi:10.1016/j.celrep.2019.11.026)
Supplement: 2 [file NIHMS1546469-supplement-2.zip › DF1/PXD006675/LeftAtrium/LeftAtrium_42_HMGB1_ENILACPLVMLR.pdf]

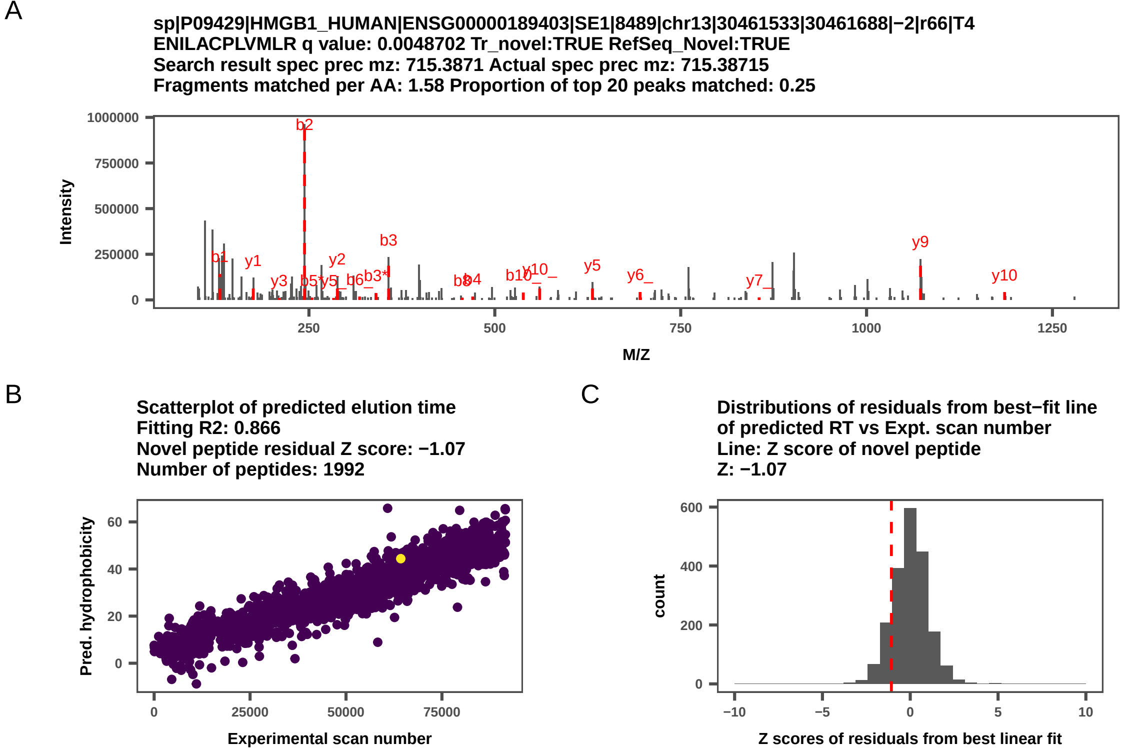
A
sp|P09429|HMGB1_HUMAN|ENSG00000189403|SE1|8489|chr13|30461533|30461688|−2|r66|T4
ENILACPLVMLR q value: 0.0048702 Tr_novel:TRUE RefSeq_Novel:TRUE
Search result spec prec mz: 715.3871 Actual spec prec mz: 715.38715
Fragments matched per AA: 1.58 Proportion of top 20 peaks matched: 0.25
Intensity
M/Z
B
Scatterplot of predicted elution time
Fitting R2: 0.866
Novel peptide residual Z score: −1.07
Number of peptides: 1992
Pred. hydrophobicity
Experimental scan number
C
Distributions of residuals from best−fit line
of predicted RT vs Expt. scan number
Line: Z score of novel peptide
Z: −1.07
count
Z scores of residuals from best linear fit
